# Supplementary figures and images for: Synchrotron‐source micro‐x‐ray computed tomography for examining butterfly eyes
Source: Ecol Evol. 2024 Apr 1;14(4):e11137. doi: 10.1002/ece3.11137 (PMC10985371; doi:10.1002/ece3.11137)

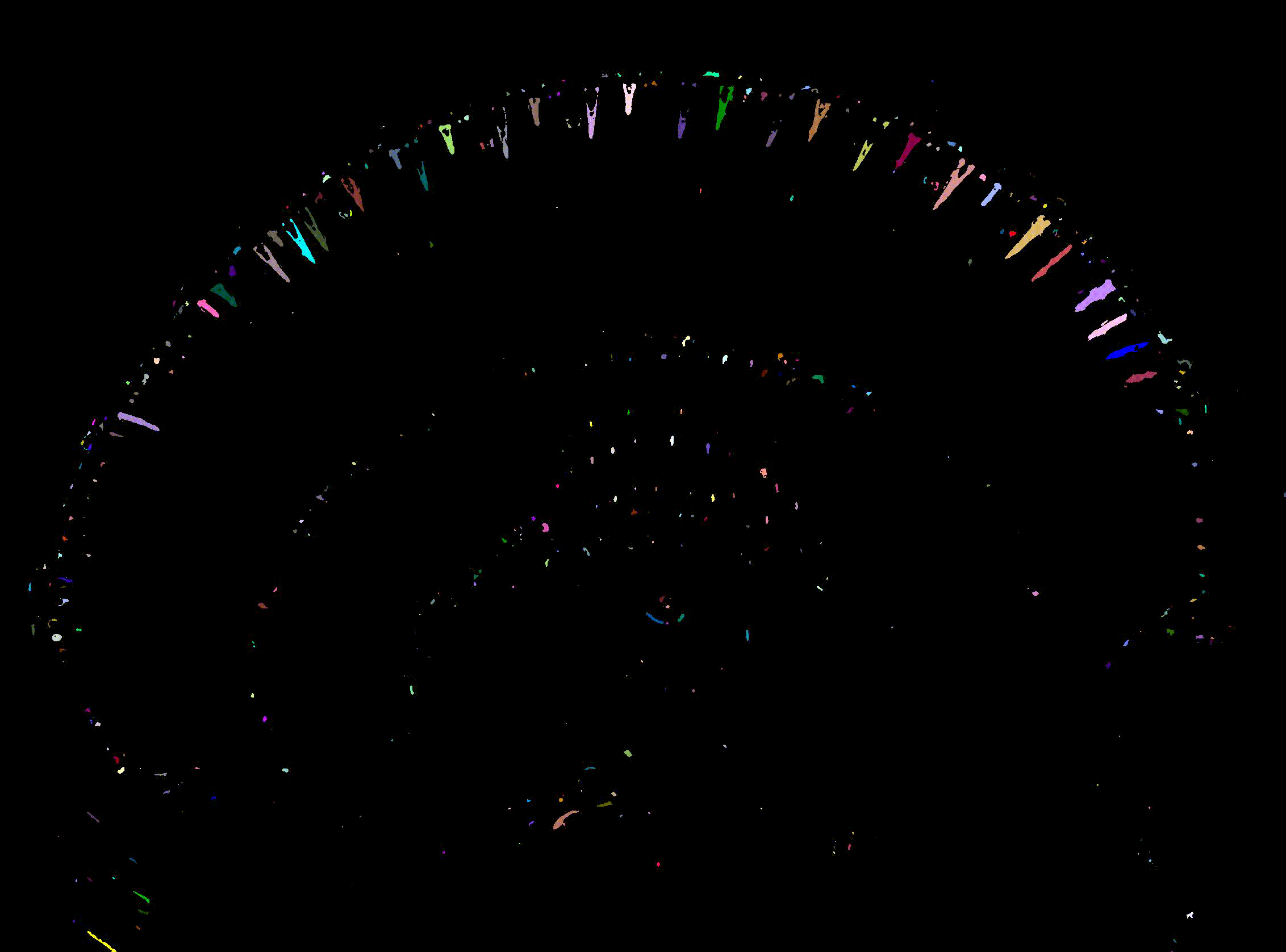

Supplement: Supplementary file 1 — Figure S1. [file ECE3-14-e11137-s003.zip › FigSI1B.gif]

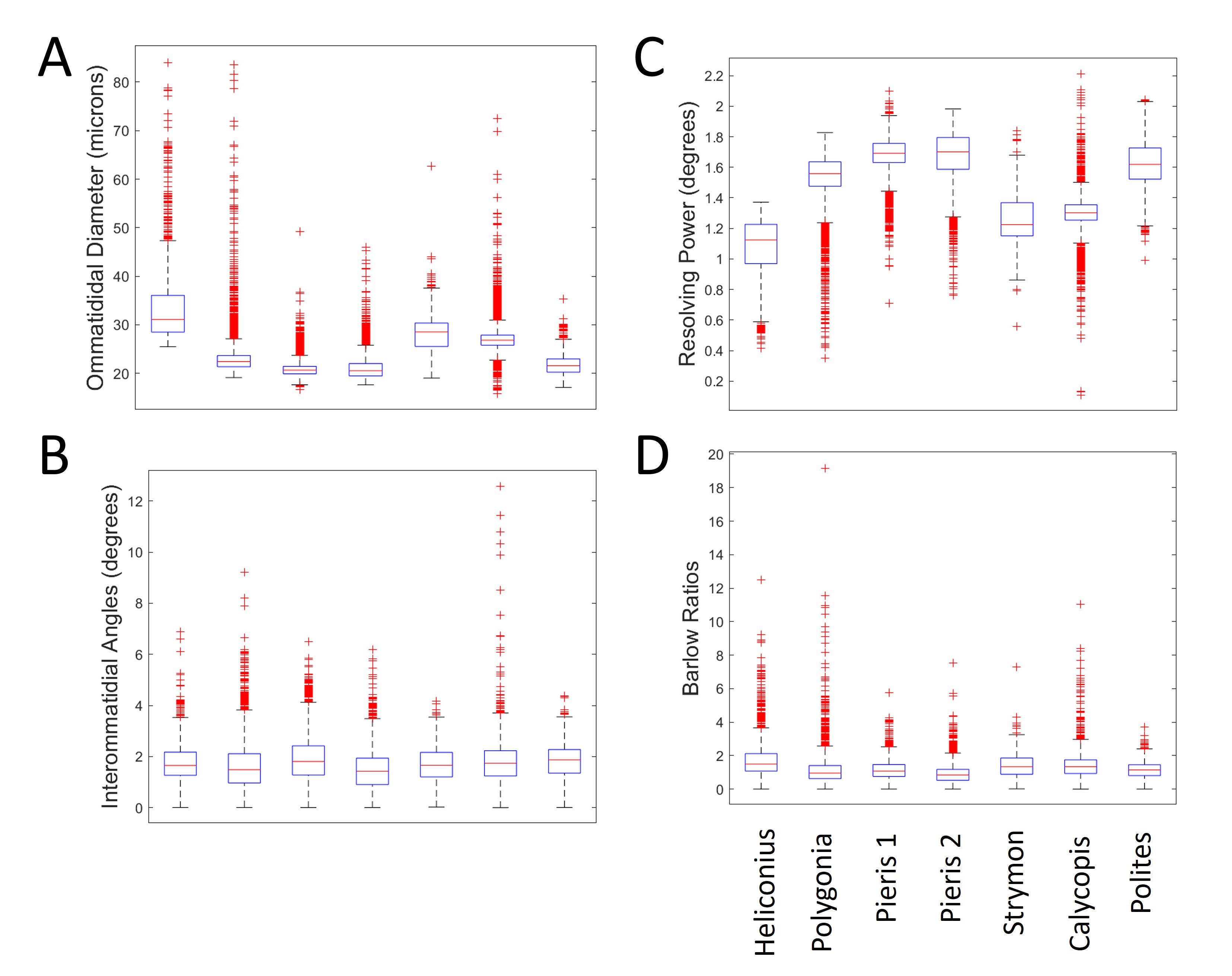

Supplement: Supplementary file 2 — Figure S2. [file ECE3-14-e11137-s002.jpg]

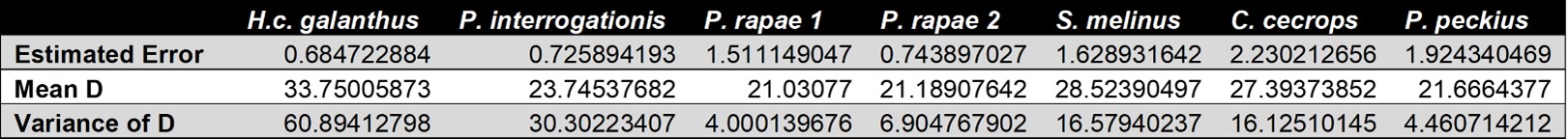

Supplement: Supplementary file 3 — Table S1. [file ECE3-14-e11137-s001.jpg]
